# Dataset for five recent years (2019 – 2023) agarwood essential oil research trends: A bibliometric analysis

**DOI:** 10.1016/j.dib.2024.110310

**Published:** 2024-03-11

**Authors:** Zakiah Mohd Yusoff, Nurlaila Ismail, Siti Aminah Nordin

**Affiliations:** a College of Engineering, Universiti Teknologi MARA, 81750 Pasir Gudang, Johor, Malaysia; b School of Electrical Engineering, College of Engineering, Universiti Teknologi MARA, 40450, Shah Alam, Selangor, Malaysia

**Keywords:** Bibliographic dataset, Agarwood, Research trends, Literature analysis

## Abstract

Research on Agarwood Essential Oils (AEO) has undergone dynamic evolution, propelled by its diverse applications in industries such as perfumery, pharmaceuticals, and alternative medicine. The aromatic richness and therapeutic potential of these essential oils have sparked a surge in research interest. Despite extensive exploration, there is a need for a comprehensive analysis of trends, patterns, and the impact of AEO research to provide insights for future studies and applications.This work presents a meticulously curated dataset encompassing the last five years of Agarwood Essential Oil (AEO) research trends. Sourced from two reputable scholarly databases, namely Web of Science and Scopus, following the Preferred Reporting Items for Systematic Reviews and Meta-Analyses (PRISMA) protocol, analyzing the data using Biblioshiny, and spanning the period from 2019 to 2023, the dataset is designed to facilitate a comprehensive understanding of the evolving landscape of AEO studies. It covers a wide array of parameters, including authorship, subject areas, citations, source titles, wordcloud, and keywords. This dataset is made available to researchers, institutions, and decision-makers to provide insights into the academic debates on agarwood oil studies, allowing for a nuanced understanding of the progression of scholarly endeavors within the field. The dataset aims to serve as a valuable resource for researchers, policymakers, and industry stakeholders interested in the multifaceted applications of essential oils. The structured and comprehensive nature of the dataset makes it a valuable asset for exploring historical trends, identifying key contributors, and fostering collaborative initiatives within the AEO research domain.

Specifications Table

**Table:** 

Subject	Agricultural and Biological Science
Specific subject area	Bibliometrics Analysis
Data format	Raw, Filtered, Analysed (in Excel)
Type of data	Excel files (dataset with numbers and labels) Table (dataset with numbers and labels) Figure
Data collection	The compilation of raw data entailed consolidating outcomes from analogous searches conducted on both the Scopus and Web of Science databases. Merging and deduplication processes were facilitated using Bibliometrix software and Excel. Adhering to the Reporting Items for Systematic Reviews and Meta-Analyses (PRISMA) protocol, which includes stages such as identification, screening, eligibility and inclusion, and the application of inclusion and exclusion criteria, articles underwent sorting via keyword reviews, abstract assessments, and, if necessary, a full article examination. A total of 249 eligible documents resulted from this process, paving the way for subsequent bibliometric analysis.
Data source location	The gathered data is sourced from the digital repositories Scopus and Web of Science, specifically targeting research articles written in English within the time frame spanning 2019 to 2023. Institution: Universiti Teknologi MARA, Johor Branch, Pasir Gudang Campus. Country: Malaysia Electronic database: Scopus https://www.scopus.com/home.uri Web of Science https://www.webofscience.com
Data accessibility	Data are with the article. Repository name: Mendeley Data Data identification number: 10.17632/pptsj7kkk8.1 Direct URL to data: https://data.mendeley.com/datasets/pptsj7kkk8/1

## 1 Value of the Data

• The dataset provides a comprehensive analysis of trends, patterns, and the impact of AEO research, offering valuable insights. Researchers can leverage this information to identify gaps in current knowledge, explore emerging areas, and guide the direction of future studies. This knowledge can inform product development, innovation, and strategic planning within these sectors.
• Policymakers can utilize the dataset to inform decisions related to regulations, funding allocation, and support for research initiatives. It provides a basis for understanding the significance and impact of AEO research within the broader context of essential oils.
• The dataset serves as a valuable resource for academic debates on agarwood oil studies. Researchers can use it to identify key contributors, explore collaborative opportunities, and engage in informed discussions within the academic community.
• The structured and comprehensive nature of the dataset enables exploration of historical trends in AEO research. This historical perspective is valuable for tracing the evolution of the field, understanding its trajectory, and predicting potential future developments.

## 2 Background

Bibliometric indicators offer a valuable method for assessing and analyzing research literature, enabling the examination and exploration of various themes and disciplines through the study of production patterns, trends, and publication impact [1,2]. This article has three primary objectives: firstly, to employ a methodological approach based on the PRISMA framework for conducting a literature review; secondly, to generate bibliometric datasets using specifically focused on the topic of agarwood essential oil; and ultimately, to present these datasets in the form of graphical visualizations using Biblioshiny. The findings from this paper serve as an initial foundation, marking the beginning of further exploration and content analysis regarding the applications of agarwood studies.

## 3 Data Description

**Fig. 1:**
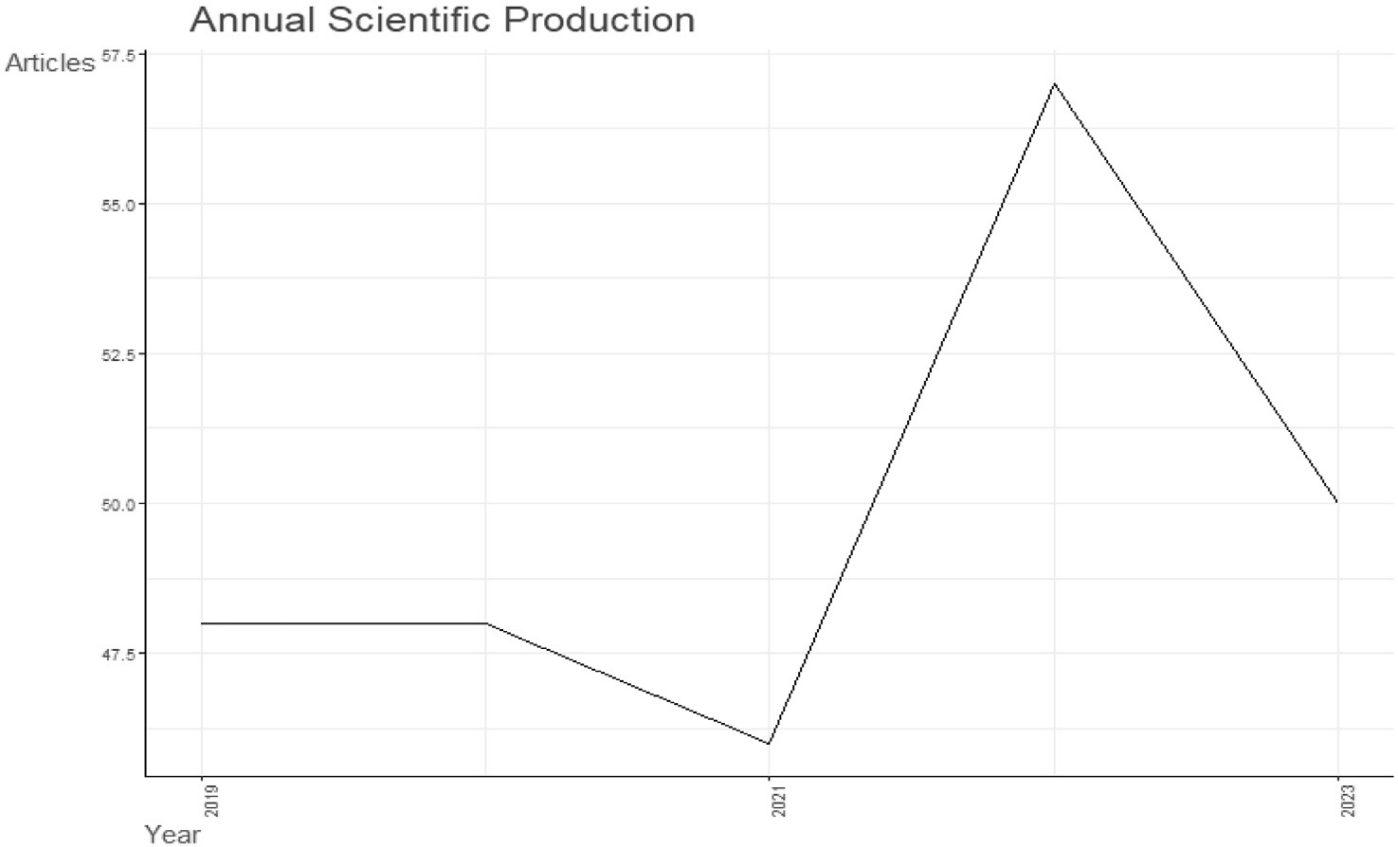
Annual scientific production.

**Fig. 2:**
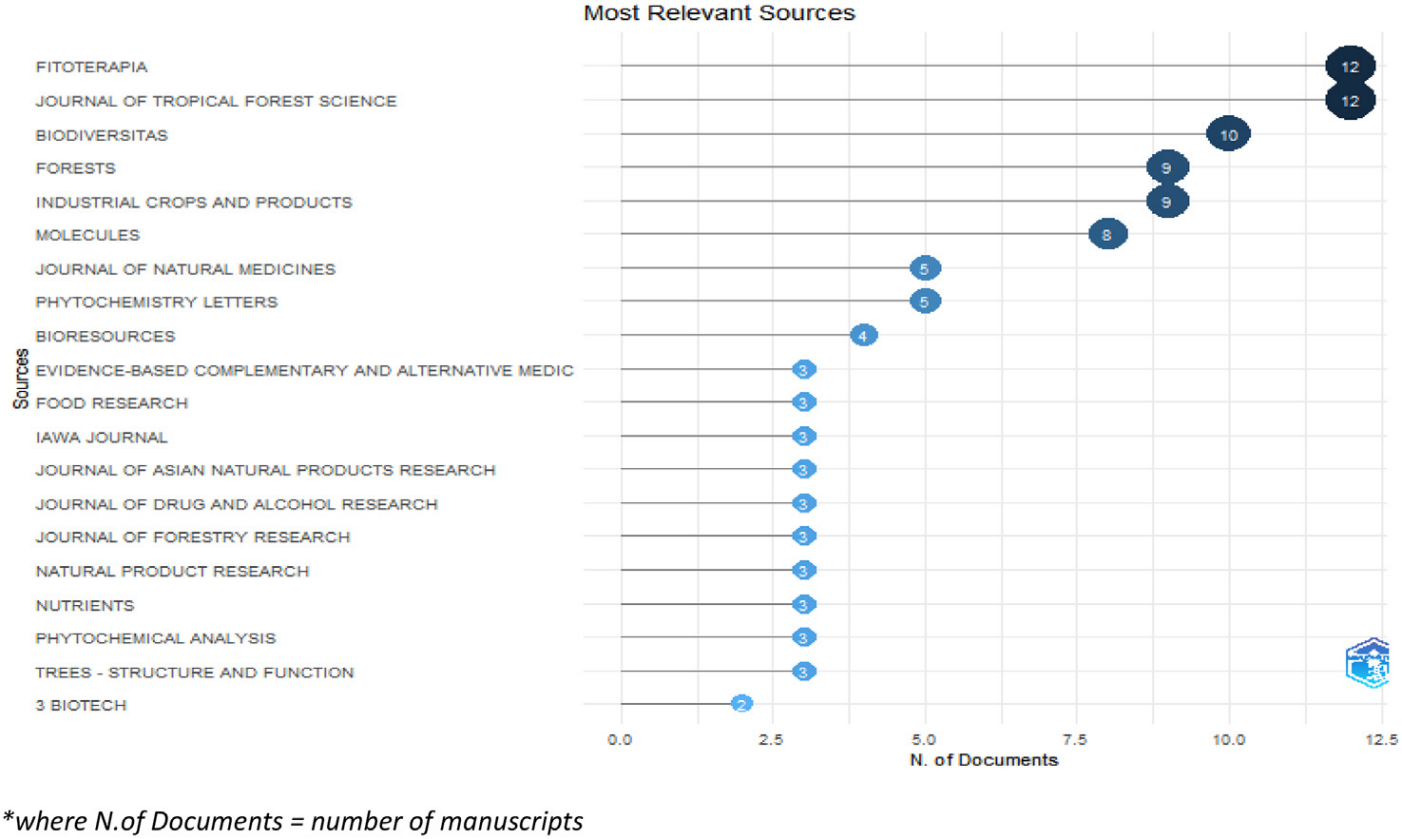
Most relevant sources.

**Fig. 3:**
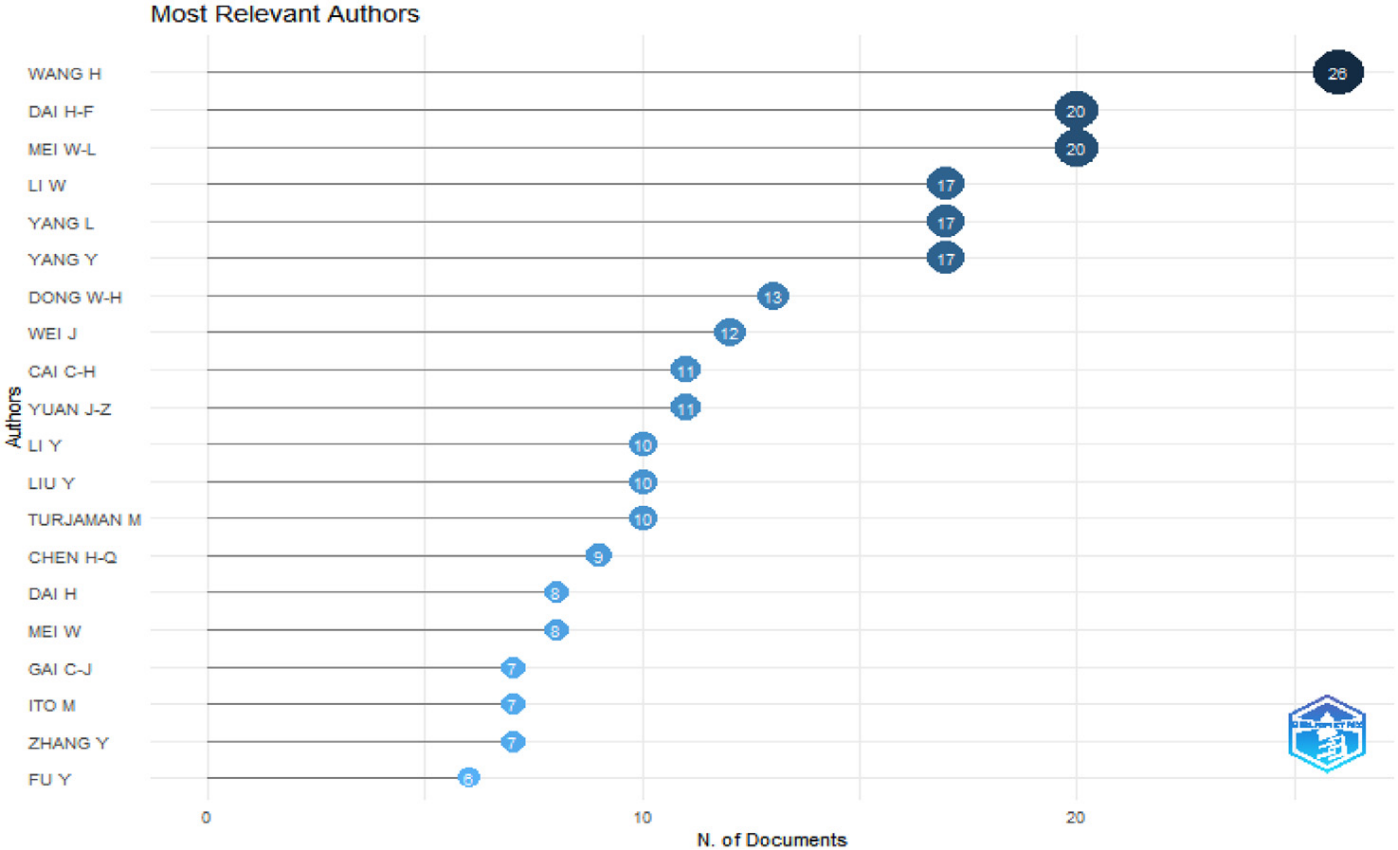
Most relevant authors.

**Fig. 4:**
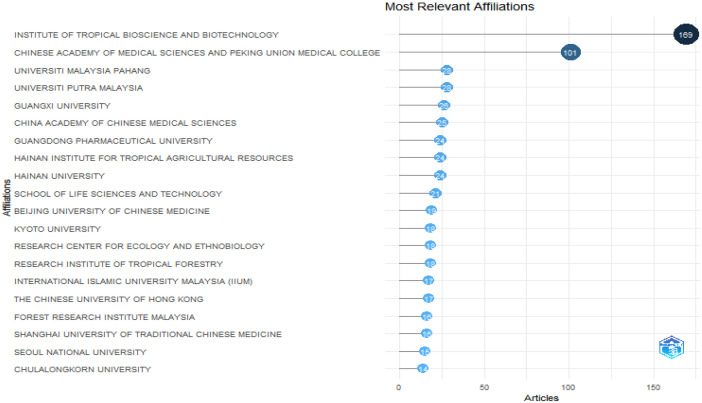
Most relevant affiliations.

**Fig. 5:**
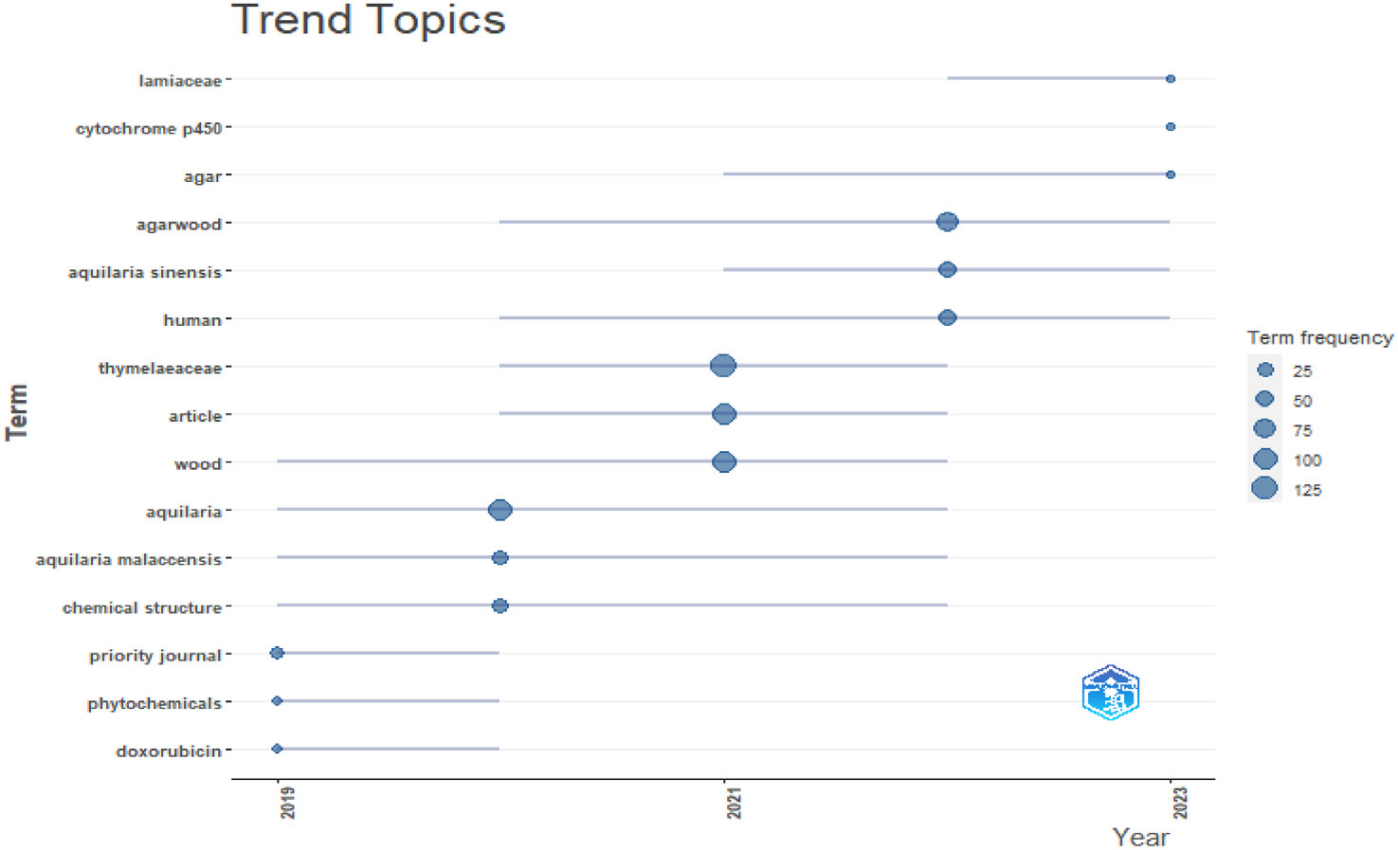
Trend topics.

**Fig. 6:**
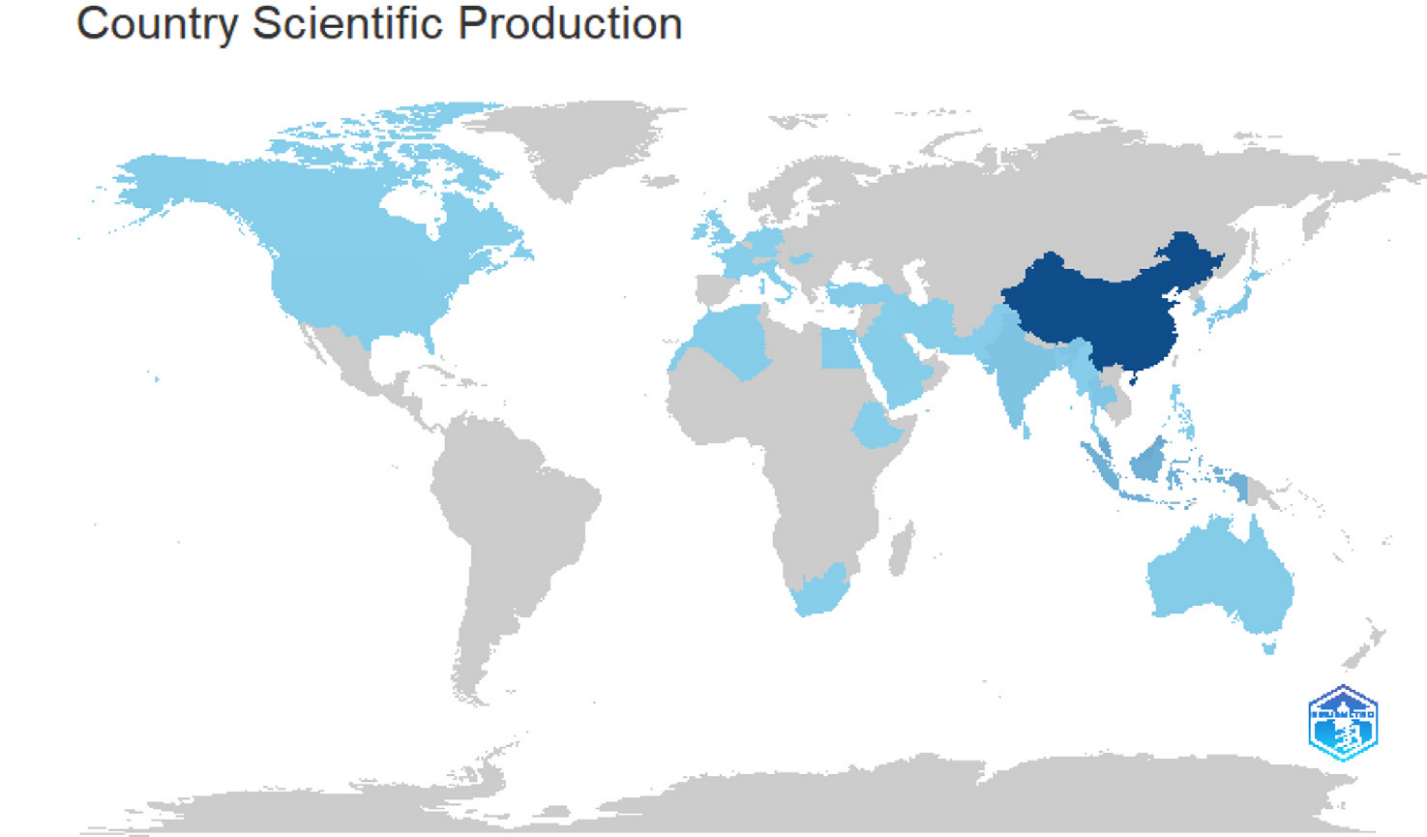
Country scientific production.

**Fig. 7:**
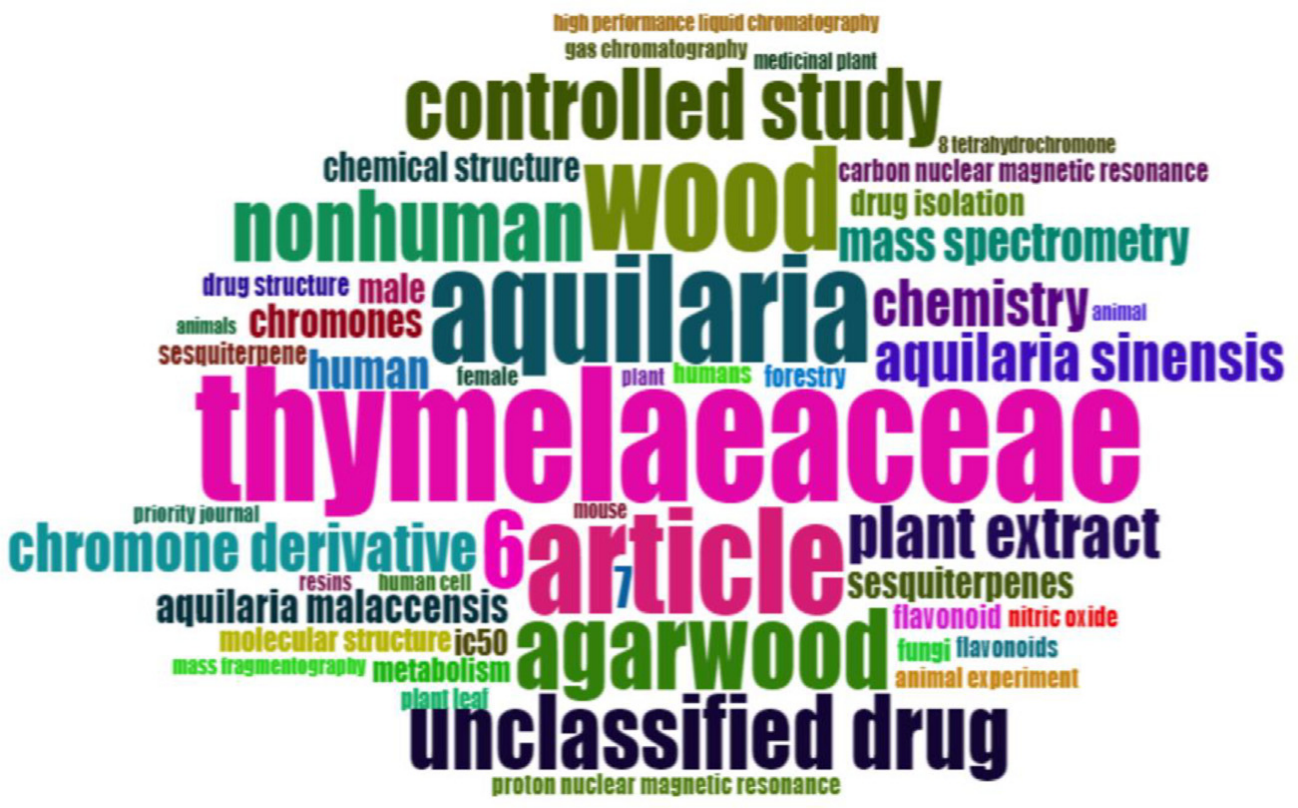
WordCloud for keywords.

**Fig. 8:**
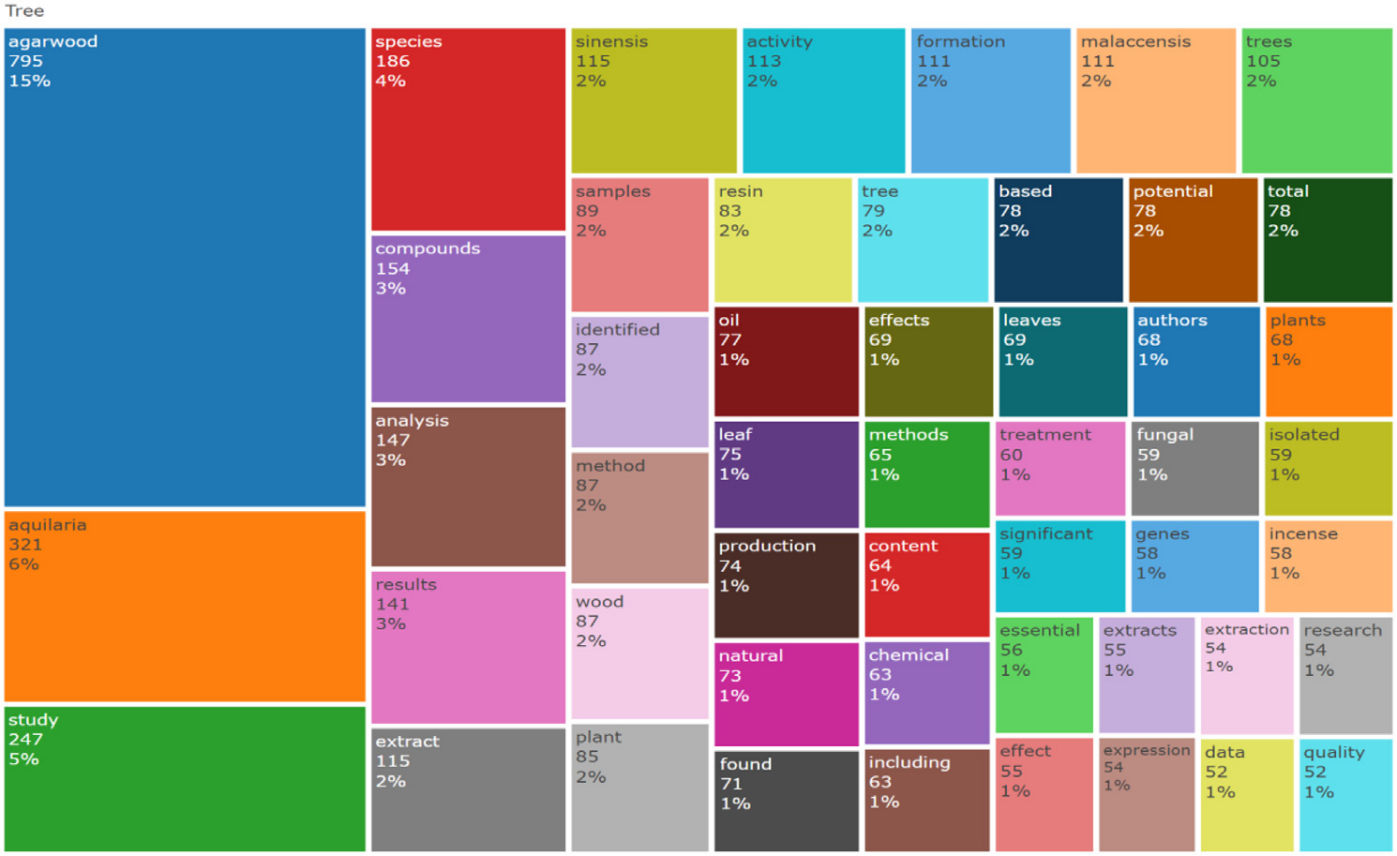
Treemap visualization of dominant title words in abstracts.

The folder named ``DATASET'' consists of two Excel files titled ``1-Bibliometrix Dataset'' and ``2-Biblioshiny Report,'' respectively. The file ``1-Bibliometrix Dataset'' is a single sheet that compiles information related to 249 articles written in English. These articles are exclusively derived from the scientific databases Scopus and Web of Science (Wos), covering the recent years of the research study (2019–2023). The metadata column encompasses 16 descriptive variables as follows: AU (Author), TI (Title), PY (Publication Year), SO (Journal), VL (Volume), Page.start, Page.end, PP (Publication Page), TC (Total Citation), Affiliations, AB (Abstract), DE (Keywords), ID (Keywords Plus), PU (Publisher), ISSN, LA (Language), and Publication.Stage. On the other hand, the ``2-Biblioshiny Report'' file contains data on MissingData, AnnualSciProd, MostRelSources, MostRelAuthors, MostRelAffiliations, MostCitCountries, CountrySciProd, WordCloud, TrendTopics, and TreeMap Top Topic in Abstract, as illustrated in Fig. 1, Fig. 2, Fig. 3, Fig. 4, Fig. 5, Fig. 6, Fig. 7, Fig. 8. Each figure is presented along with its corresponding data in the table.

Fig. 4 illustrates the affiliations most commonly associated with research on Agarwood Essential Oils (AEO), extracted from the Biblioshiny software. Each node represents an affiliation, with the size indicating the frequency of affiliation occurrences and the color gradient reflecting the geographical distribution. Data extraction involved identifying affiliations listed in scholarly articles retrieved from the Scopus and Web of Science databases, spanning the years 2019 to 2023. Darker shades on the color gradient represent regions with higher frequency of affiliations Tables 1 and 2.

**Table 1:** Top 10 country scientific publication.

Region	No. of manuscripts
China	776
Malaysia	210
Indonesia	166
India	57
Thailand	50
South Korea	45
Japan	42
Bangladesh	18
Sri Lanka	17
Australia	14

**Table 2:** Keywords frequency.

Terms	Frequency
Thymelaeaceae	132
Aquilaria	105
Article	104
Wood	103
Agarwood	81
6	74
Unclassified Drug	74
Controlled Study	71
Nonhuman	71
Plant Extract	53
Aquilaria Sinensis	49
Chromone Derivative	49
Chemistry	46
7	41
Mass Spectrometry	39
Human	38
Aquilaria Malaccensis	34
Chromones	33
Sesquiterpenes	31

## 4 Experimental Design, Materials and Methods

### 4.1 Identification

The systematic review process employed in the selection of a considerable number of pertinent publications for this investigation comprised four crucial stages. In the first stage, we chose keywords and explored synonymous terms using thesauri, dictionaries, encyclopedias, and prior research. Subsequently, by formulating search strings for the Scopus and Web of Science databases (refer to Table 3), we identified all relevant keywords. In the initial phase of the procedure, a total of 2447 publications were successfully compiled for the ongoing study project from both databases.

**Table 3:** The search string.

Scopus	TITLE-ABS-KEY (agarwood OR gaharu OR aquilaria) AND (LIMIT-TO (PUBYEAR, 2019) OR LIMIT-TO (PUBYEAR, 2020) OR LIMIT-TO (PUBYEAR, 2021) OR LIMIT-TO (PUBYEAR, 2022) OR LIMIT-TO (PUBYEAR, 2023)) AND (LIMIT-TO (DOCTYPE, ``ar'')) AND (LIMIT-TO (PUBSTAGE, ``final'')) AND (LIMIT-TO (LANGUAGE, ``English'')) AND (LIMIT-TO (SRCTYPE, ``j'')) AND (LIMIT-TO (EXACTKEYWORD, ``Agarwood'') OR LIMIT-TO (EXACTKEYWORD, ``Aquilaria''))
Web of Science (Wos)	agarwood OR gaharu OR aquilaria (Topic) and 2023 or 2022 or 2021 or 2020 or 2019 (Publication Years) and Article (Document Types) and English (Languages)

### 4.2 Screening

In the screening phase, the collection of potentially relevant research materials undergoes a thorough examination to identify content that aligns with the predefined research questions. Criteria associated with the content, commonly applied during this screening phase, include agarwood, aquilaria and gaharu when selecting research items. This phase also entails the removal of all duplicate papers from the initially retrieved list. The initial screening resulted in the exclusion of 1799 publications, while the subsequent phase involved assessing 648 papers based on distinct inclusion and exclusion criteria outlined in this study (refer to Table 4). The primary criterion for inclusion was literature in the form of research papers, serving as the main source for practical recommendations. This encompassed reviews, meta-synthesis, meta-analyses, books, book series, chapters, and conference proceedings not considered in the most recent study. Additionally, the review exclusively focused on English-language publications, underscoring that the analysis concentrated on the years 2019 to 2023. In total, 176 publications were excluded due to duplication criteria.

**Table 4:** The inclusion and exclusion criteria.

Criterion	Inclusion	Exclusion
Language	English	Non-English
Timeline	2019 – 2023	< 2019
Literature type	Journal (Article)	Conference, Book, Review
Publication Stage	Final	In Press

### 4.3 Eligibility

**Fig. 9:**
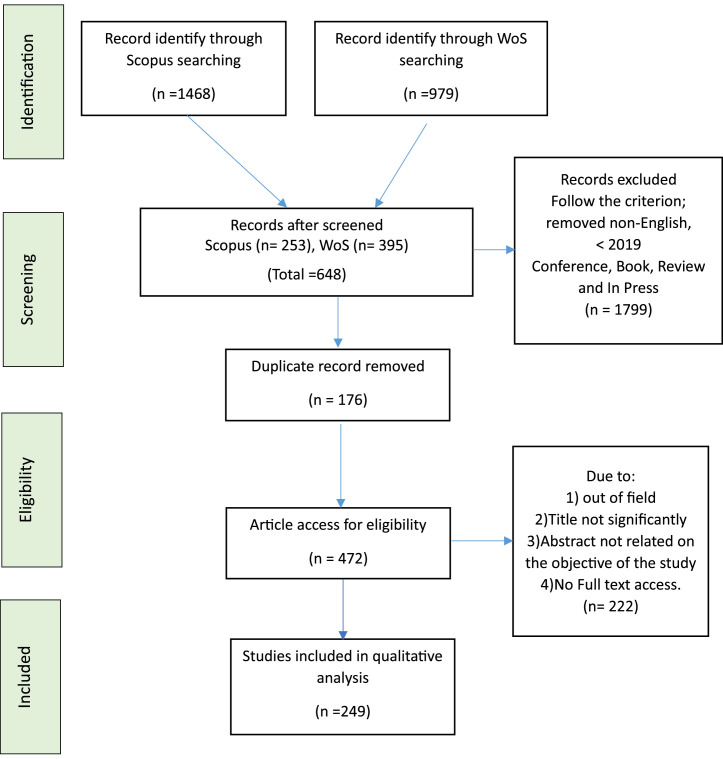
Flow diagram of the PRISMA framework [5].

In the third phase, referred to as eligibility, a set of 472 articles was gathered. This stage entailed a thorough scrutiny of the titles and core content of all articles to determine their conformity with the inclusion criteria and their relevance to the present research objectives. As a result, 222 reports were excluded for various reasons, including being beyond the study scope, lacking in significance, containing abstracts unrelated to the study's objective, and lacking full-text access or empirical evidence to support the content. In the end, 249 articles fulfilled the criteria and are now ready for review, as shown in Fig. 9.

### 4.4 Inclusion

In this study, integrative analysis was employed as a key assessment strategy, aiming to thoroughly examine and integrate various research designs, with a primary emphasis on quantitative methods. The objective of this comprehensive examination was to pinpoint relevant topics and subtopics. The initial stage of theme development focused on collecting data within the agarwood oil domain, as depicted in Fig. 9. This process resulted in a collection of 249 documents for bibliometric analysis. The assessment utilized the Biblioshiny [3,4] web-based package application, acknowledged for its effectiveness in bibliometric analysis, citation metric analysis, and network mapping.

## Limitations

Not applicable.

## Ethics Statement

The authors have read and follow the ethical requirements for publication in Data in Brief and confirming that the current work does not involve human subjects, animal experiments, or any data collected from social media platforms.

## CRediT authorship contribution statement

**Zakiah Mohd Yusoff:** Conceptualization, Writing – review & editing. **Nurlaila Ismail:** Resources, Validation, Methodology. **Siti Aminah Nordin:** Formal analysis.

## Data Availability

Dataset for Five Recent Years ( 2019 – 2023) Agarwood Essential Oil Research Trends: A Bibliometric Analysis (Original data) (Mendeley Data).
